# Revealing suitable habitats for *Juniperus procera* and *Olea europaea* tree species in the remnant dry Afromontane forests of Ethiopia: Insights from ensemble species distribution modeling approach

**DOI:** 10.1002/ece3.70343

**Published:** 2024-10-03

**Authors:** Gebreyohannes Zenebe, Amanuel Zenebe, Emiru Birhane, Atkilt Girma, Henok Shiferaw

**Affiliations:** ^1^ Institute of Climate and Society (ICS) Mekelle University Mekelle Ethiopia; ^2^ Department of Land Resource Management and Environmental Protection, College of Dryland Agriculture and Natural Resources Mekelle University Mekelle Ethiopia; ^3^ Faculty of Environmental Sciences and Natural Resource Management Norwegian University of Life Sciences (NMBU) Ås Norway

**Keywords:** Desa'a forest, habitat suitability, Hugumbirda Grat‐Kahsu forest

## Abstract

Human activities and climate change pose a significant threat to the dry Afromontane forests in Ethiopia, which are essential for millions of people both economically and ecologically. In Ethiopia, trees are planted elsewhere even if they are not likely to be well suited to the area. This study aims to identify the suitable habitat for the most exploited *Juniperus procera* (*J. procera*) and *Olea europaea* (*O. europaea*) tree species in northern Ethiopia. As inputs, least correlated temperature, moisture, soil, and topographic variables were selected through a stepwise procedure. The study evaluated five individual and ensemble models using the area under the curve (AUC) and true skill statistic (TSS) values. The ensemble model outperformed with mean AUC of 0.95 and TSS of 0.78 for *J. procera*, while securing the second position for *O. europaea* with an AUC of 0.88 and TSS of 0.71. Climatic factors emerged as the most influential, followed by soil and topography. Suitable areas for both species were found when Isothermality (Bio3) values range from 52% to 62%, temperature seasonality (Bio4) of 16–29°C. Moreover, well drained soils with soil texture not heavier than sandy clay, and soil organic carbon ranging from 5 to 42 g kg^−1^ were found suitable. The optimal suitable altitude for *J. procera* and *O. europaea* was determined to be 2200–2600 and 2100–2500 m.a.s.l., respectively. The suitable areas for *J. procera* and *O. europaea* were estimated to be 3130 and 3946 km^2^, respectively. Furthermore, potential plantation areas were identified beyond Desa'a and Hugumbirda Grat‐Kahsu protected forests, covering 2721 km^2^ (86.9%) for *J. procera* and 3576 km^2^ (90.6%) for *O. europaea*. These findings hold significance for the conservation and sustainable management of these valuable tree species in northern Ethiopia. We recommend implementing a similar approach for other locally restricted dry Afromontane tree species with wider potential distribution.

## Graphical abstract

The study aims to identify suitable habitats for the most exploited *Juniperus procera* and *Olea europaea* tree species in northern Ethiopia, using temperature, moisture, soil, and topographic variables. It evaluates five individual and ensemble models using area under the curve and true skill statistic values. The ensemble model performed well for *J. procera* and *O. europaea*, with climatic factors being the most influential. Suitable areas for both species were found in areas with Isothermality values between 52% and 62%, temperature seasonality between 16 and 29°C. The optimal altitude for *J. procera* and *O. europaea* is 2200–2600 and 2100–2500, with suitable areas of 3130 and 3946 km^2^, respectively. Potential plantation areas are identified beyond protected forests, promoting conservation and sustainable management.

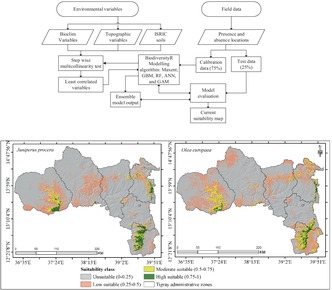

## 1 INTRODUCTION

The largest extent of the Afromontane environment in the world is found in Ethiopia (Young et al., 2017), and dry Afromontane forests form the largest part (Worku & Soromessa, 2015). These forest ecosystems have immense socio‐economic, cultural, and ecological benefits for the local community. Additionally, dry Afromontane forests have the potential to store huge amounts of carbon in their biomass and soil (Solomon et al., 2018). Despite their indispensable importance, these forest ecosystems are among the most altered and threatened (Siyum et al., 2019; Tura et al., 2017), and are constantly shrinking, primarily as a result of anthropogenic pressures (Gelashe, 2017). The combined effect of climate, topographic and local human disturbance factors controlled the stability of the dry Afromontane forest in Ethiopia (Hishe et al., 2021). In Ethiopia, climate change affected the sustainability of numerous indigenous tree species (Gufi et al., 2023).

Dry Afromontane forest restoration is a top priority in Ethiopia and a global priority for ecosystem restoration (Fisseha & Rannestad, 2022). Ethiopia has been adopting many mitigating actions that attempt to restore forest resources. As part of its national green growth strategy and to support the New York Declaration on Forests and the Bonn Challenge, the Ethiopian government committed in 2014 to restore 15 million hectares of degraded and deforested landscapes by 2025 (MEFCC, 2017). The Ethiopian Government had launched a massive country‐wide afforestation campaign called Green Legacy Initiative (GLI) in 2018. For this to succeed, the whole country was mobilized and engaged in tree planting and millions of seedlings were claimed to be freely distributed. However, the survival rate and hence performance of GLI may not be as high as what is reported by the government due to less attention given to post‐planting activities (Beyene & Shumetie, 2023; Takele et al., 2022), and a lack of proper identification of suitable habitats for planting specific trees among others. To derive the expected economic, social, and environmental benefits from the GLI, environmental factors such as climatic condition, soil moisture, rainfall, and temperature need to be considered in selecting trees for planting (Abebe & Arega, 2023).

In the Tigray region, dry Afromontane forests are found as remnant forests within either church forests, protected state forests, and less accessible areas (Aerts et al., 2006). Desa'a and Hugumbirda Grat‐Kahsu are the two national forest priority areas in Tigray‐Ethiopia, despite patchy regional protected forest areas. In addition to their socio‐economic importance, the Desa'a and Hugumbirda Grat‐Kahsu remnant dry Afromontane forests have ecological importance as a buffer in protecting the spread of the desert to the highlands of Tigray region (Berihu et al., 2017). The dominant species in the two forest remnants are *Olea europaea* and *Juniperus procera*. However, *J. procera* and *O. europaea* subsp. *cuspidata* are undergoing a dramatic retreat (tree line shifts) and degradation, and being replaced by a lowland dominant economically less valued species (Hishe et al., 2015; Woldemichael et al., 2010). In Desa'a forest alone, *J. procera* and *O. europaea* lost nearly 40% and 33% of their area between 1972 and 2013, respectively (Hishe et al., 2015). *Juniperus procera* is included on the International Union for Conservation of Nature's (IUCN) red list of endangered species (Tigabu et al., 2007). Because of its multipurpose use, which causes overexploitation, *O. europaea* is also locally endangered in Ethiopia (Wegasie et al., 2021). *Olea europaea* wood is commonly used to make farm equipment and furniture, as well as for fencing, fuelwood, and making charcoal. Both the leaves and the roots are also used for traditional smoke baths and medicine (Ourge et al., 2018), its branches are used as toothbrush stick. The importance of *J. procera* and *O. europaea* for fuelwood, timber, traditional medicine, and non‐timber forest products in East Africa has also been reported in a study by Jones (2015). Restoring these species would contribute to biodiversity conservation and enhance the livelihood of the rural population.

Over the past few decades, large tracts of degraded land in northern Ethiopia, particularly in Tigray, have been protected from uncontrolled human and domestic animal interference in an effort to slow down the alarming rate of deforestation and rehabilitate severely degraded landscapes. As a result, the vegetation cover of the region has increased to 11% (Hagazi et al., 2020), though the cover is decreased by 5% due to the war on Tigray (Negash et al., 2023). Yet, restoration of the degraded dry Afromontane forests in Northern Ethiopia through exclosures requires a better understanding of the ecological requirements of the different species (Aynekulu et al., 2009). Trees are planted elsewhere in Ethiopia when ecological suitability for the area of interest or the interests of local stakeholders are not appropriately taken into account during selection (Reubens et al., 2011). Understanding tree species response to environmental factors and human disturbance is important for developing sustainable management and conservation plans (Walthert & Meier, 2017). Without such knowledge, management guidelines assisting the restoration process have limitation on implementing appropriate management (Aerts et al., 2006). Evidence‐based restoration options for degraded ecosystems in Ethiopia's Afromontane highlands are scarce (Abiyu et al., 2017). Species distribution models are essential tools for mapping suitable habitats, providing reliable, dependable, and repeatable information to inform decisions.

A study by Gastón et al. (2014) has pointed out the suitability of a species distribution modeling approach to obtain fast and cost effective recommendations for matching species to sites in forest restoration projects. Different researchers use distinct species distribution models (SDMs) to make decisions about the conservation of endangered plants (Abrahms et al., 2017; Wang et al., 2015). Girma et al. (2015) and Abrha et al. (2018), also modeled the suitable habitat of *Boswellia* and *J. procera*, respectively, using MAXENT model in northern Ethiopia. However, the selection of one algorithm over another could lead to different results and therefore to different conclusions (Montoya‐Jiménez et al., 2022). To reduce a single model's uncertainty, several other ensemble models need to be employed. It is suggested that the ecological niche models need to be species‐specific, carefully selected, and combined/ensemble (Mcintyre et al., 2017). Ensemble modeling generated with several statistical methods has been recommended to improve predictions of the ecological range of a species (Grenouillet et al., 2011; Kindt, 2018). Comparing the predictive power of different modeling techniques, ensemble modeling generally performs better than individual modeling techniques (Engler et al., 2013). The accuracy of biodiversity conservation and management in remnant forests may be improved using ensemble techniques (Porfirio et al., 2014). Ensemble models are primarily used to reduce the generalization error in prediction.

To the best of our knowledge, no study has been published to date that linked the remnant dry Afromontane tree species' current geographic locations with the least correlated temperature, moisture, soil, and topographic variables, using an ensemble modeling framework to determine their suitable habitat in the area. Thus, the study was aimed at identifying potential suitable areas for *J. procera* and *O. europaea*, two dominant characteristic tree species, conservation in northern Ethiopia; evaluating the predictive accuracy of ensemble modeling to that of individual models; and identifying the range of environmental variables for *J. procera* and *O. europaea* suitability determination.

## 2 MATERIALS AND METHODS

### 2.1 Study area and species

**FIGURE 1:**
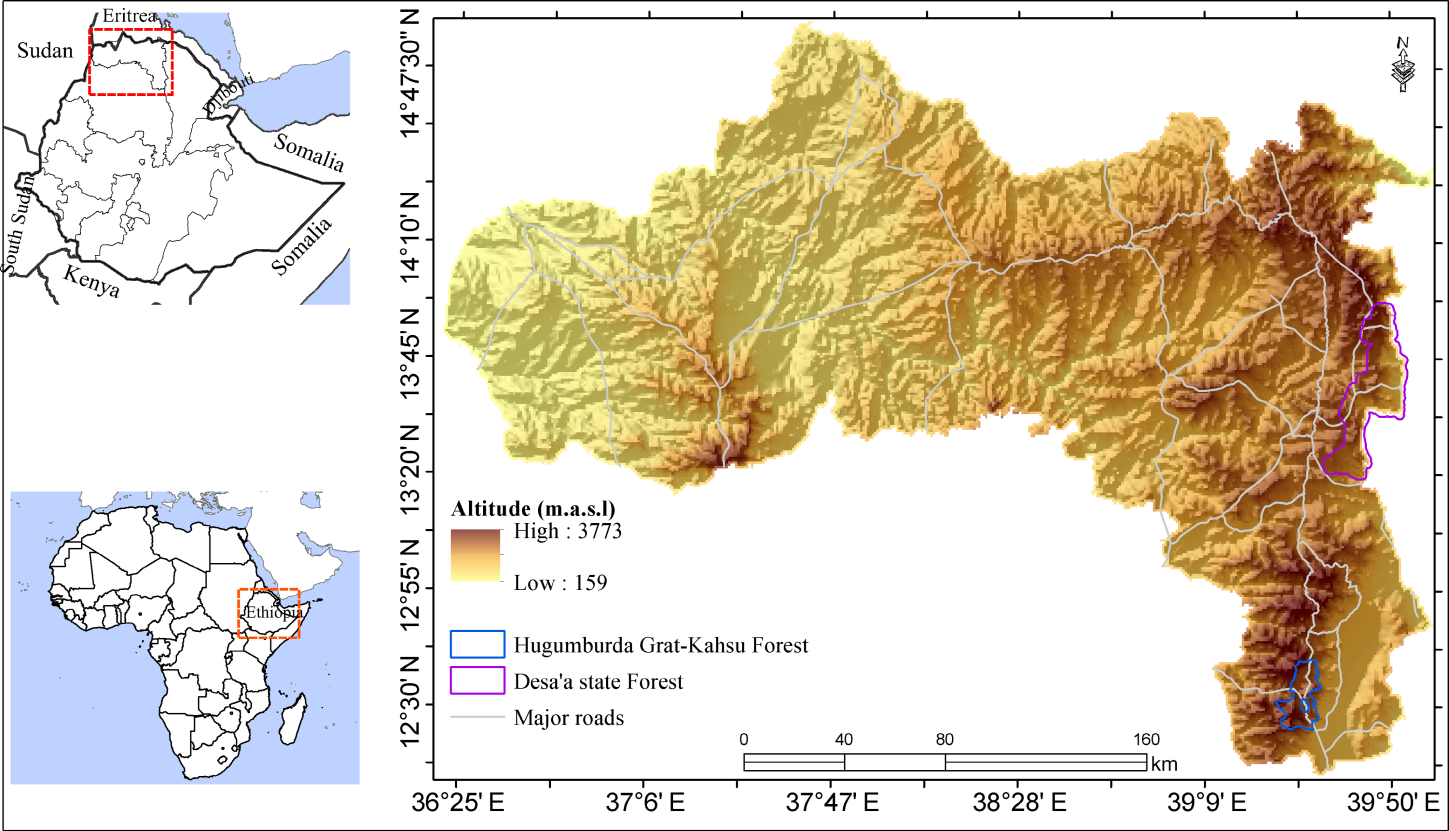
Location of the study area.

The study area encompasses the entire Tigray regional state, Northern Ethiopia. Tigray region is located between 12°15′ N and 14°57′ N latitude and 36°27′ E and 39°59′ E longitude (Figure 1). The region covers 53,000 km^2^, with an altitude ranging from 159 to 3773 m above the sea level. The region's agro‐climatic classification is primarily hot semi‐arid, warm semi‐arid, tepid semi‐arid, and hot arid, covering 90.8% of the area. Cool semi‐arid, tepid arid, and warm arid agro‐climatic areas contribute 6.7%, while other zones have less than 2.5% coverage (Hagos et al., 2021). The region's climate is predominantly semi‐arid, with the main rainy season occurring from mid‐June to early September and the warmest season from March to May.

**FIGURE 2:**
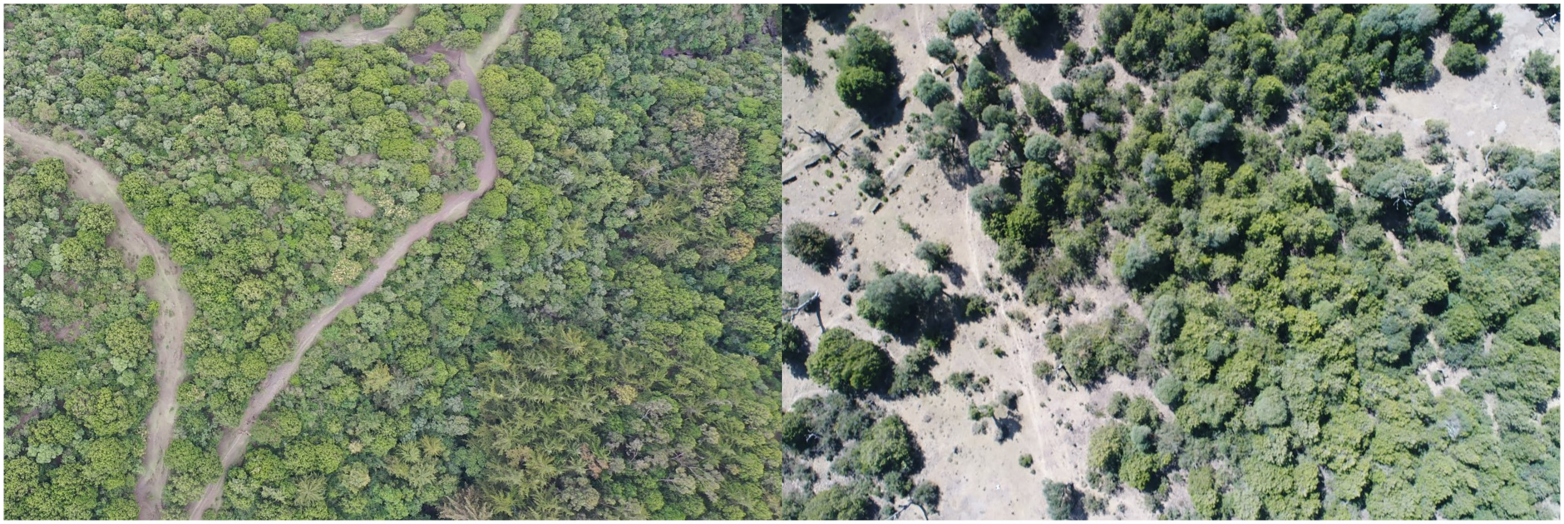
Aerial unmanned aerial vehicle photograph captured during field visit at Hugumbirda Grat‐Kahsu (left) and Desa'a (right). Photos: Gebreyohannes Zenebe.

Desa'a and Hugumbirda Grat‐Kahsu are the two national forest priority areas in the region (Figure 1), and are categorized as dry Afromontane forests with *J. procera* and *O. europaea subsp. cuspidata* as the dominant species (Friis, 1992; Figure 2). Desa'a forest is found in the eastern part of Tigray region. The level of degradation in the Desa'a forest is severe where the dominant species are declining up along the altitude gradient (Aynekulu et al., 2012). Among the woody species in the Desa'a forest, *O. europaea* and *J. procera* are the most exploited (Giday et al., 2013). The Hugumbirda Grat‐Kahsu national forest priority area is also found in the southern part of Tigray Region. There are four districts bordering the forest: Alamata, Endamehoni, Ofla, and Raya Azebo. It was designated as a national forest priority area in 1993 (Woldemichael et al., 2010). The forest is dominated by small‐sized tree and shrub species in a secondary stage of development.

**FIGURE 3:**
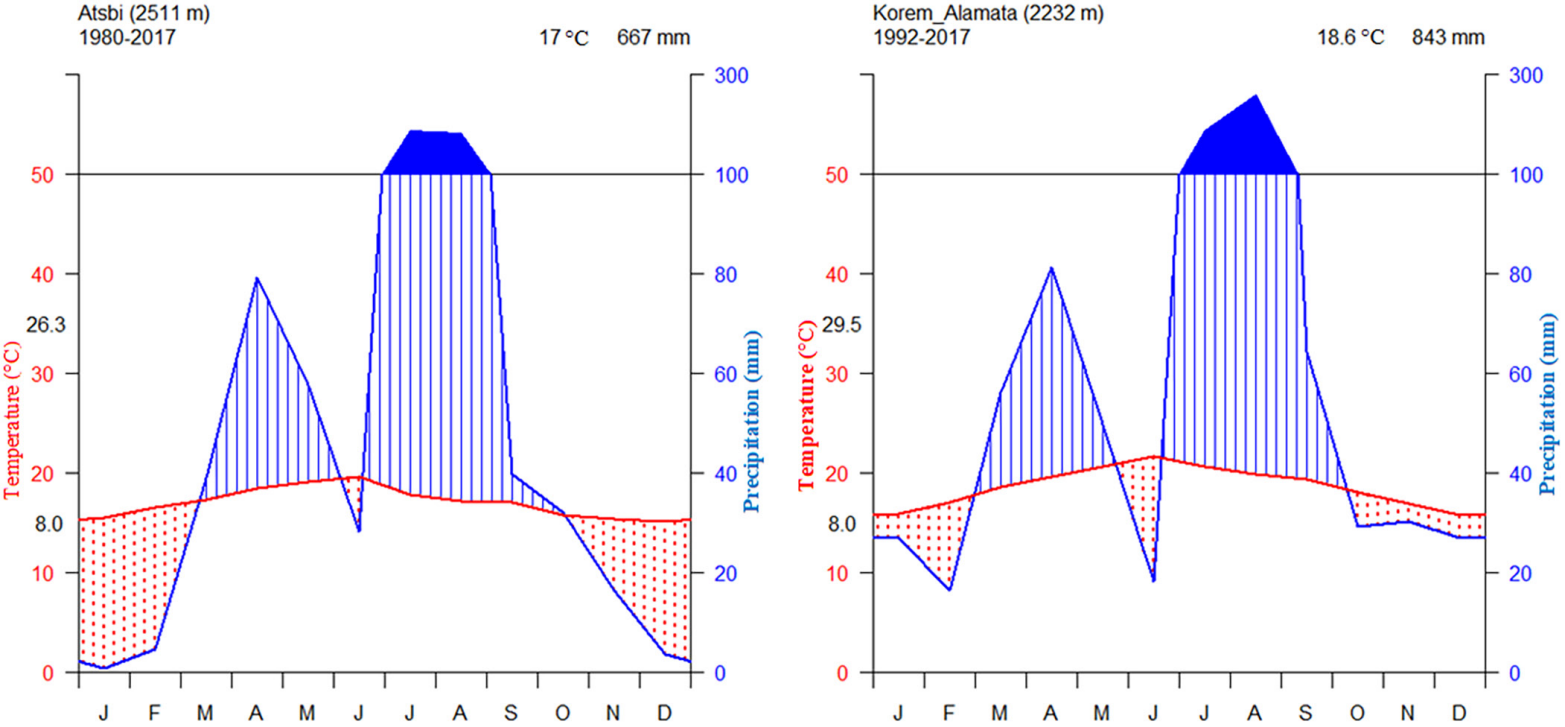
Climate diagram for Atsbi (left) Korem‐Alamata (right): The left axis of the diagram shows temperature from 0 to 50°C, while the right axis shows precipitation from 0 to 100 mm. The temperature curve is shown in red, and the precipitation curve is shown in blue. The area with blue shaded lines illustrates the humid conditions below the threshold, whereas the area dotted in red indicates the arid conditions. The area filled in blue shows the humid conditions above the threshold.

A climate diagram was plotted to categorize climate situations and to show moisture conditions (wet, dry, humid periods) for Atsbi and Korem‐Alamata meteorological stations, adjacent to Desa'a and Hugumbirda Grat‐Kahsu, respectively (Figure 3).

As per the Atsbi meteorological station data obtained from the National Meteorological Agency of Ethiopia for the years 1980–2017, the average daily maximum temperature of the hottest month for Atsbi is represented by the value at the top left of the temperature axis (26.3°C), while the average daily minimum temperature of the coldest month is represented by the value at the bottom of the same axis (8°C) (Figure 3, left). The mean annual temperature and rainfall of the Atsbi meteorological station was 17°C and 667 mm, respectively. The average daily minimum and maximum temperature of the hottest month for the Korem‐Alamata meteorological stations from 1980 to 2017 ranges between 8 and 29.5°C, respectively (Figure 3, right). And the average annual temperature and rainfall was 18.6°C and 843 mm, respectively.

### 2.2 Datasets

#### 2.2.1 Species location data

This study focused on the two most important but degraded characteristic indigenous tree species of *J. procera* and *O. europaea*. During the fieldwork, spatially distributed presence and absence records were collected from Desa'a and Hugumbirda Grat‐Kahsu forests and their surroundings using a handheld global positioning system. The georeferenced locations were meticulously checked for duplications and typos to minimize potential errors in the modeling process. Finally, 350 and 168 presence, and 209 and 148 absence locations with a distance between them not larger than 2 km for *J. procera*, and *O. europaea*, respectively, were used. All points at which the species was not detected during the survey period were used as absences. Finally, the presence and absence records were arranged separately in an excel format with three columns corresponding species name, longitude, and latitude coordinates.

#### 2.2.2 Environmental variables

For this purpose, 1 km resolution time series climate data (1980 to 2016) were downloaded from the Climatologies at high resolution for the Earth's land surface areas (CHELSA) database (Karger et al., 2017). The 37 years climate data were then averaged to the monthly mean of all the years. Consequently, 19 bioclim variables were generated using dismo package biovars function in R v 3.5.3 software (R Core Team, 2019). We also incorporated soil variables accessed from the ISRIC's 250 m resolution SoilGrids database (Hengl et al., 2017). Six soil variables (Bulk Density, Cation Exchange Capacity, Depth to bedrock, Soil pH, Soil Organic Carbon, and Soil Texture) were used. Additionally, we considered topographic variables as an input in the modeling process (Altitude, slope gradient, and aspect) derived from the Shuttle Radar Topography Mission (SRTM) digital elevation model (DEM) 30 m resolution accessed from the USGS Global Visualization Viewer/EarthExplorer (http://earthexplorer.usgs.gov/).

Finally, from the aforementioned input datasets, a total of 28 predictor variables (19 bioclim, 6 soil variables, and 3 topography) were produced and processed to have the same extent, projection system, and grid resolution as the DEM 30 m resolution in R software.

### 2.3 Multicollinearity test and variable selection

The selection of environmental variables to be used in species distribution modeling is an important aspect of the modeling process. Prior to model calibration, all the variables were subjected to a multicollinearity test to determine which ones were the most useful and non‐redundant. A study by Worthington et al. (2016) has confirmed that these highly relevant variables introduce redundant information into the model prediction process affects the prediction results. The variance inflation factor (VIF) (Naimi et al., 2014), from the car package in R was used to evaluate multicollinearity. A VIF value that exceeds 10 indicates a problematic amount of collinearity (James et al., 2013). From the initial 28 predictor variables, 15 variables with VIF less than 10 or a tolerance level of above 0.1 were selected through a stepwise procedure, that were deemed statistically and biologically meaningful to model the suitable habitat distribution of *J. procera* and *O. europaea* (Table 1).

**TABLE 1:** Least correlated environmental layers used to model the habitat suitability of study species.

Code	Description	Unit	Correlation test	
			Variance inflation factor	Tolerance level
Altitude	Elevation	m.a.s.l.	4.01	0.25
Bio3	Isothermality (bio2/bio7) × 100	%	4.06	0.25
Bio4	Temperature seasonality (standard deviation × 100)	°C	3.47	0.29
Bio8	Mean temperature of wettest quarter	°C	5.68	0.18
Bio12	Annual precipitation	mm	2.81	0.36
Bio14	Precipitation of the driest month	mm	3.43	0.29
Bio15	Precipitation seasonality (coefficient of variation)	mm	2.35	0.43
Bio18	Precipitation of warmest quarter	mm	2.25	0.44
BLD	Bulk density	g m^−3^	4.26	0.23
CEC	Cation exchange capacity	meq 100 g^−1^	4.07	0.25
DBR	Depth to bedrock	Cm	1.27	0.79
PH	Soil pH	‐	3.19	0.31
Slope	Slope gradient	% rise	1.54	0.65
SOC	Soil organic carbon	g kg^−1^	1.83	0.55
Texture	Soil texture	finer to coarser	3.83	0.26

### 2.4 Modeling procedure

To obtain an ensemble of predicted suitable habitat for *J. procera* and *O. europaea*, a total of five widely used algorithms were implemented through the graphical user interface of the BiodiversityR package version 2.11‐3 (Kindt & Coe, 2005), generated by the R‐Commander: four machine learning algorithms, Maximum Entropy (Maxent) (Hijmans et al., 2017), Generalized Boosted Regression Modeling (GBM/BRT) (Greenwell et al., 2019), Random Forest (RF) (Liaw & Wiener, 2002), Artificial Neural Network (ANN) (Venables & Ripley, 2002); one regression method, generalized additive model (GAM) (Hastie, 2019). Maxent is a presence‐only (and background) data model, while the other four models use presence and absence data as input. To calibrate the models, and to evaluate their predictive performance, 4‐fold cross‐validation runs were carried out by randomly splitting 75% of the data for calibration and the remaining 25% for testing. We used both the area under the curve (AUC) of the receiver operating characteristic curve and the true skill statistic (TSS) to measure model performance. AUC and TSS metrices have been used in several studies (Khan & Verma, 2022; Wani et al., 2022). Modeling algorithms with AUC of 0.7 and above were set to create the final ensemble model. AUC is considered as an effective way to evaluate model performance since it is a single, independent measure with values ranging from 0 to 1 (Allouche et al., 2006). TSS offers the advantage of improving the overall model accuracy and is a relatively new measure to evaluate a model's predictive performance (Bedia et al., 2011). Sensitivity and specificity are alternative techniques for model evaluation derived from the confusion matrix (Equation 1).

(1) $$\mathrm{TSS}=\frac{\mathrm{ad}-\mathrm{bc}}{\left(a+c\right)\left(b+d\right)}=\text{Sensitivity}+\text{Specifity}-1.$$

TSS takes into account both sensitivity which measures omission errors and specificity which measures the commission error, and ranges from −1 to +1, where +1 indicates perfect agreement and values of zero indicates no better than random, and less than zero worth than random (Allouche et al., 2006). TSS is not affected by prevalence and the size of the validation set, and that two methods of equal performance have equal TSS scores.

### 2.5 Model evaluation and analysis

**FIGURE 4:**
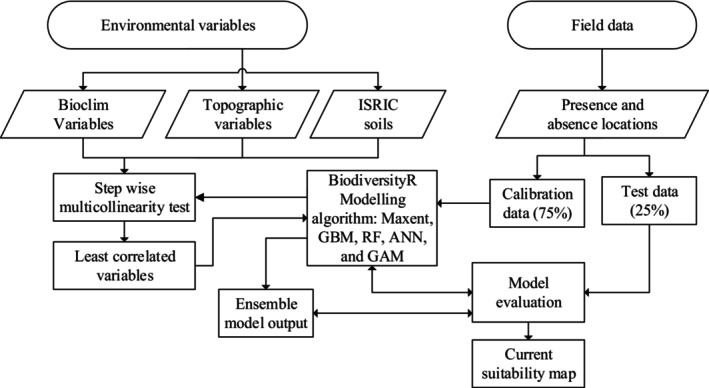
Schematic workflow used for the study. ANN, Artificial Neural Network; GAM, generalized additive model; GBM, Generalized Boosted Regression Modeling; RF, Random Forest.

In this study, the reliability and validity of the individual algorithms and the ensemble model based on AUC values were graded as invalid (AUC <0.6), bad (0.6–0.7), acceptable (0.7–0.8), good (0.8–0.9), and excellent (≥0.9) as used by (Thuiller et al., 2008). TSS values were also evaluated as poor (<0.4), fair (0.4–0.6), good (0.6–0.8), and excellent (>0.8) using the TSS classification defined by González‐Ferreras et al. (2016). From the four replications and five modeling algorithms, we obtained a single average model evaluation output and an ensemble of predicted distributions for both species. Each of the five modeling methods and an ensemble model produced an output map of continuous values between zero and one corresponding to the probability that a pixel is suitable habitat or not for the species. Finally, the potential suitable habitat maps for the ensemble and each of the five model outputs were converted into binary (habitat vs. no habitat) maps using the four probability classes defined by Shrestha and Bawa (2014): unsuitable (<0.25), low potential (0.25–0.5), medium potential (0.5–0.75) and high potential (>0.75). Area calculation and percentage coverage for each classification were also performed. The schematic workflow for the overall methods used in this study is presented in Figure 4.

## 3 RESULTS

### 3.1 Model performance evaluation

The results of the two metrics used to assess model accuracy (AUC and TSS values) for the ensemble and single models 4‐fold cross‐validation run and their mean are shown in Tables 2 and 3, respectively. The modeling process is repeated four times, and each of the folds is used once as the testing dataset. The results obtained with the testing data from all four runs are then averaged to produce a single estimation. The statistical evaluation of individual models and ensemble output yielded varying values in predictive performance statistics. The mean AUC value of the ensemble model for *J. procera* was the highest (0.95) followed by GBM (0.94), GAM (0.93), and MAXENT (0.92). The GBM performed slightly better with an AUC value of 0.89 for *O. europaea* followed by the ensemble (0.88), GAM (0.87), MAXENT (0.87), and RF (0.87) (Table 2). ANN's AUC values were the lowest for both species compared to other algorithms, yet still within the range of good evaluation metrics (AUC >0.8).

**TABLE 2:** Test area under the curve for each 4‐fold cross‐validation run and their mean of the algorithms used to model the suitable habitat of *Juniperus procera* and *Olea europaea.*

Models	*J. procera*					*O. europaea*				
	T_1	T_2	T_3	T_4	Mean	T_1	T_2	T_3	T_4	Mean
ENSEMBLE	0.93	0.95	0.95	0.96	0.95	0.84	0.92	0.87	0.90	0.88
GBM	0.93	0.94	0.95	0.94	0.94	0.85	0.93	0.88	0.90	0.89
GAM	0.94	0.92	0.94	0.92	0.93	0.83	0.92	0.85	0.87	0.87
MAXENT	0.91	0.90	0.92	0.94	0.92	0.83	0.92	0.85	0.87	0.87
RF	0.90	0.89	0.86	0.92	0.89	0.84	0.94	0.80	0.91	0.87
ANN	0.90	0.88	0.90	0.81	0.87	0.79	0.92	0.74	0.79	0.81

Abbreviations: ANN, Artificial Neural Network; GAM, Generalized Additive Model; GBM, Generalized Boosted Regression Modeling; Maxent, Maximum Entropy; RF: Random Forest.

**TABLE 3:** True skills statistic for each 4‐fold cross‐validation run and their mean of the algorithms used to model the suitable habitat of *Juniperus procera* and *Olea europaea.*

Models	*J. procera*					*O. europaea*				
	T_1	T_2	T_3	T_4	Mean	T_1	T_2	T_3	T_4	Mean
ENSEMBLE	0.76	0.78	0.77	0.79	0.78	0.66	0.80	0.67	0.71	0.71
GBM	0.73	0.74	0.77	0.76	0.75	0.60	0.82	0.71	0.70	0.71
GAM	0.77	0.70	0.79	0.74	0.75	0.59	0.83	0.71	0.70	0.71
MAXENT	0.71	0.67	0.66	0.78	0.70	0.68	0.66	0.66	0.66	0.66
RF	0.75	0.75	0.70	0.82	0.76	0.60	0.81	0.61	0.69	0.68
ANN	0.66	0.70	0.75	0.60	0.68	0.60	0.82	0.39	0.60	0.60

Abbreviations: ANN, Artificial Neural Network; GAM, Generalized Additive Model; GBM, Generalized Boosted Regression Modeling; Maxent, Maximum Entropy; RF, Random Forest.

Like that of AUC, the mean TSS value of the ensemble model for *J. procera* was the highest (0.78) followed by RF (0.76), GBM (0.75), and GAM (0.75) (Table 3). The mean TSS value of ensemble output, GBM, and GAM for *O. europaea* performed approximately equal (0.71) (Table 3). The TSS values of ANN were the lowest among other algorithms, yet still within a range of good evaluation metrics (TSS >0.6).

### 3.2 Response of the species to environmental variables

To understand the effect of predictor variables on the prediction results, the inter‐quartile potential range of the top contributing predictor variables to the ensemble output is shown in Table 4. Both *J. procera* and *O. europaea* are found when precipitation of driest month (Bio14) ranges from 2 to 17 mm. *J. procera* and *O. europaea* tree species occurred in areas with temperature seasonality (Bio4) ranging from 16 to 26 and 18 to 29°C, respectively. The mean temperature of the wettest quarter (Bio8) ranging from 11 to 24 and 11 to 28°C was found to be suitable for *J. procera* and *O. europaea* tree species, respectively. Areas are also suitable for both species when Isothermality (Bio3) values range from 52% to 62%. Moreover, well‐drained soils with soil texture not heavier than sandy clay (3), soil organic carbon (SOC) ranging from 5 to 42 g kg^−1^, and annual precipitation (Bio12) greater than 200 mm were found to be suitable for both species. Areas with depth to bedrock goes from 40 to 170 and 20 to 170 cm are suitable for *J. procera* and *O. europaea*, respectively.

**TABLE 4:** Potential range of environmental variables for the ensemble output.

Code	Description	Unit	Suitability range	
			*Juniperus procera*	*Olea europaea*
Bio14	Precipitation of driest month	mm	2–17	2–17
Bio4	Temperature seasonality	°C	16–26	18–29
Bio8	Mean temperature of wettest quarter	°C	11–24	11–28
Bio3	Isothermality	%	52–62	52–62
SOC	Soil organic carbon	g kg‐1	5–42	5–42
DBR	Depth to bedrock	cm	40–170	20–170
Bio12	Annual Precipitation	mm	200–1100	200–1100
BIO15	Precipitation seasonality (CV)	mm	0.6–1.6	0.6–1.6
Altitude	Elevation	m.a.s.l	2200–2600	2100–2500

### 3.3 Species potential suitable habitat maps

**FIGURE 5:**
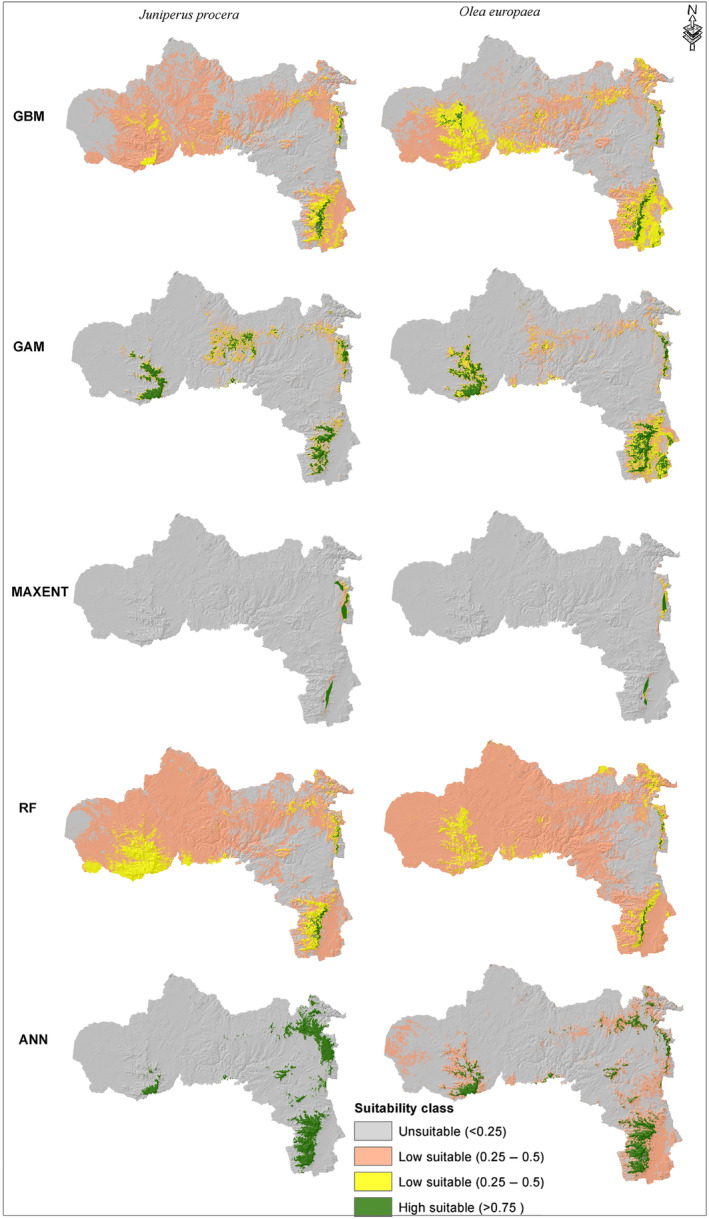
Predicted suitable habitat using five models for *Juniperus procera* and *Olea europaea*. ANN, Artificial Neural Network; GAM, Generalized Additive Model; GBM, Generalized Boosted Regression Model; Maxent, maximum entropy; RF, Random Forest.

**FIGURE 6:**
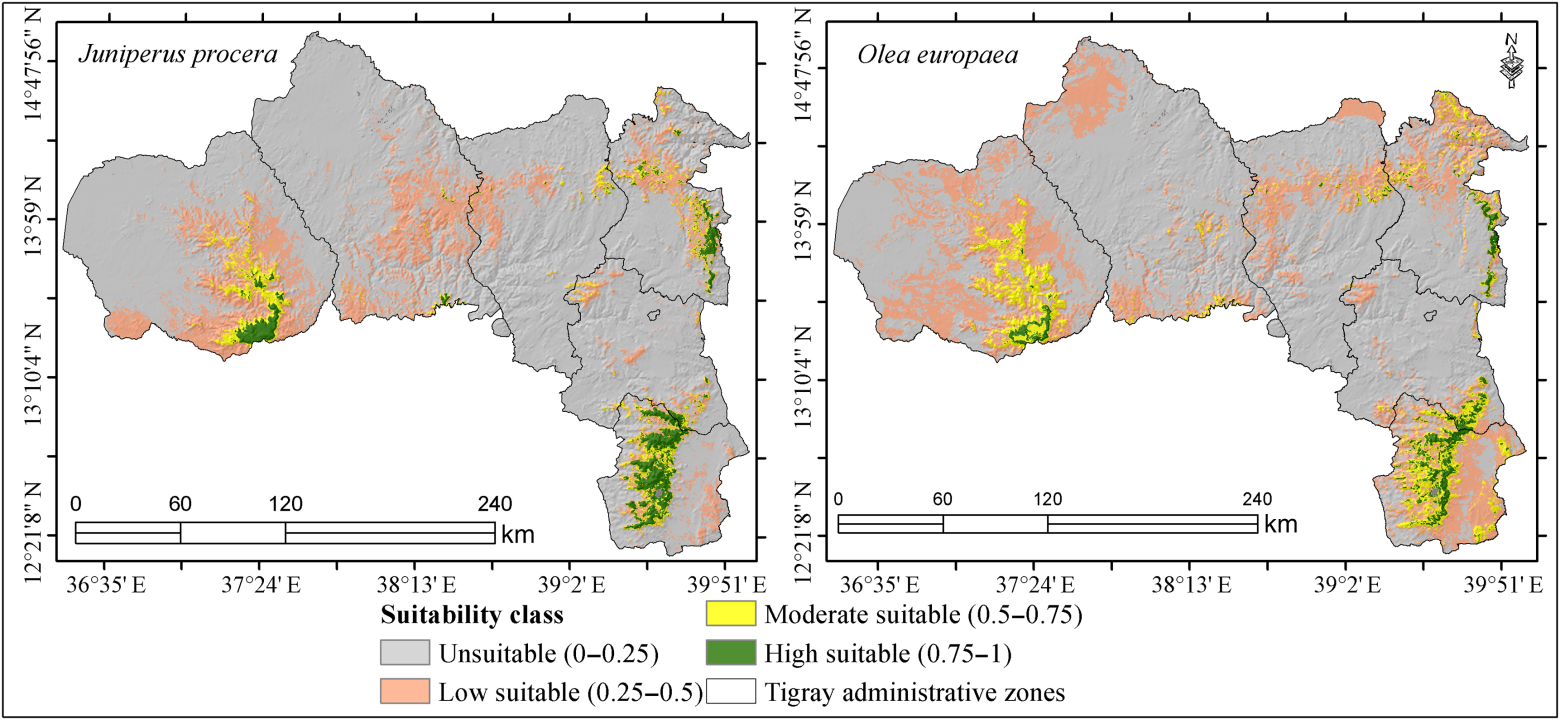
Ensemble potential suitable habitat map for *Juniperus procera* and *Olea europaea*.

The current potential suitable habitat maps of *J. procera* and *O. europaea* for individual models and ensemble outputs are presented under Figures 5 and 6, respectively. To represent the ranges of *O. europaea* and *J. procera* suitable habitat distribution, the output probability values for the individual and ensemble models were segmented into four categories: unsuitable (0–0.25), low suitable (0.25–0.5), moderate suitable (0.5–0.75), and highly suitable (0.75–1) (Figures 5 and 6). Similar to the mean AUC and TSS values, each of the ecological niche models used in this study yielded different results in spatial prediction for both species (Figure 5). The majority of individual models output for both *O. europaea* and *J. procera* predicted suitable habitat in the highlands of Southern, Eastern, and Western zones of the Tigray region. Additionally, the GAM, predicted suitable habitat for both *J. procera* and *O. europaea* in a few parts of the northwestern and central zones. Compared to other models, ANN predicted the widest highly suitable areas for both *O. europaea* and *J. procera*. On the other hand, the MAXENT model predicted the narrowest suitability, restricted to the highlands of southern and eastern zones.

From the four replications and five modeling algorithms, we also obtained an ensemble potential suitable habitat for *O. europaea* and *J. procera* tree species (Figure 6). In this way, areas of high model agreement were distinguished from areas of low model agreement. The resulting ensemble maps (Figure 6) showed integer values from 0 to 1, with a score of zero indicating that none of the modeling techniques assigned this area as suitable habitat and a value of one indicating that all modeling techniques assigned this area as suitable habitat. Though majority of the moderately to highly suitable areas for both species were found in the highlands of Southern, Eastern, and Western zones of the Tigray region, considerable suitable areas for *O. europaea* were also found at Raya Alamata district: Selen Wuha village; at Raya Azebo district: Adis Kigni, Ulaga, Korme, Hade Alga, and Horda villages; at Hintalo Wajirat district: Tsehafti, Adi keyih, Senale, and Gonka villages; at Enderta district: Lemlem and Derga'ajen villages.

The total area predicted for each species and suitability classes are also presented in Table 5. There are about 3130.4 and 3946.7 km^2^ moderately to highly suitable areas for *J. procera* and *O. europaea*, respectively, throughout Tigray. From the total estimated suitable area (above 0.5 probabilities), approximately 2721 km^2^ (87%) for *J. procera*, and 3576 km^2^ (91%) for *O. europaea* were found outside the two protected remnant forests of Desa'a and Hugumbirda Grat‐Kahsu, suggesting potential for scaling of plantations.

**TABLE 5:** Area of each habitat suitability classes for the ensemble model output of *Juniperus procera* and *Olea europaea* tree species.

Species	Potential habitat suitability class and area (km^2^)				
	Not suitable (0–0.25)	Low suitable (0.25–0.5)	Moderately suitable (0.5–0.75)	Highly suitable (>0.75)	Total suitability (>0.5)
*J. procera*	41,801.1	9046.7	1656.7	1473.7	3130.4
*O. europaea*	36,945.5	13,086.2	2956.5	990.2	3946.7

## 4 DISCUSSION

### 4.1 Model performance

The study results showed that model performance varied between the individual model algorithms and species. Due to their sensitivity to data and assumptions used to define species distribution based on environmental variables, modeling algorithms produce different predictions every time, even when trained on the same data set repeatedly. The inconsistency between the model outputs of the five algorithms used in this study for both species is consistent with other studies compared with different modeling techniques (Dittrich et al., 2020; Montoya‐Jiménez et al., 2022). As per the AUC and TSS classification by Thuiller et al. (2008) and González‐Ferreras et al. (2016), respectively, the individual and ensemble models for both species were rated from good to excellent (AUC >0.8 and TSS >0.6). The differences in the modeling techniques' performance and prediction stability in this study suggest that, the ensemble model has the potential to model a suitable habitat for the selected tree species, as also suggested by Montoya‐Jiménez et al. (2022). The evaluation metrics value to model *J. procera* identified the ensemble output as the top performing with AUC of 0.95 and TSS of 0.78. On the other hand, GBM obtained the highest performance with AUC of 0.89 and TSS of 0.71 for *O. europaea* with a slight difference from the ensemble model (AUC = 0.88 and TSS = 0.71). Our modeling result is in line with the finding of Kindt (2018), that ensemble models either had the highest AUC values or had AUC values very close to those of the best models. Yet, it is important to indicate that GBM may not always achieve the best prediction performance, as there is no single model that performs exceptionally well compared to others in all contexts (Nzuva & Nderu, 2019), An ensemble approach improves predictions by aggregating outputs from multiple models, reducing model uncertainty and sensitivity, and mitigating biases in individual SDMs, resulting in more balanced and accurate outcomes. Just as a group of individuals may make smarter decisions than one, combining the predictions from different models helps improve accuracy and reliability. It's comparable to getting advice from lots of people with different perspectives in order to make better decisions. The ensemble model leverages the strengths of various models while minimizing their weaknesses, thereby enhancing the accuracy of predictions.

### 4.2 Response of the species to environmental variables

**FIGURE 7:**
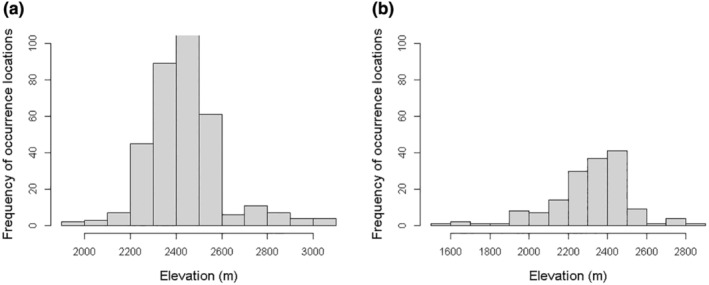
Histogram of species occurrence locations relative to elevation: *Juniperus procera* (a), *Olea europaea* (b).

Climate‐related variables followed by edaphic/soil and topographic variables were found to be important factors in determining the potential suitable habitat of *J. procera* and *O. europaea* tree species in the study area. Our result concurs with finding of Wang et al. (2003), who found that temperature is an important climatic factor for the growth of plants in semiarid or arid regions. Our finding indicates that, when isothermality range exceeds 62%, the distribution of *J. procera* and *O. europaea* tree species keeps constant. As stated by O'Donnell and Ignizio (2012), an isothermal value of 100 denotes that the diurnal temperature range is equivalent to the annual temperature range, implying that the study species can be highly affected by high temperature variations. Areas are also found to be suitable for both species when the annual precipitation ranges from 200 to 1100 mm, indicating that the species are adapted to high elevation climates with low precipitation. When rainfall exceeds 1400 mm year^−1^, *J. procera* is replaced by moister evergreen forests, making it rarer (Couralet & Bakamwesiga, 2007). Well‐drained soils with soil texture not heavier than sandy clay (3), SOC ranging from 5 to 42 g kg^−1^, and areas with depth to bedrock ranging from 40 to 170 and 20 to 170 cm are found to be suitable for *J. procera* and *O. europaea* tree species, respectively. The study finding coincides with the findings of Couralet and Bakamwesiga (2007) and van der Vossen et al. (2007), who reported that *J. procera* and *O. europaea* prefers rocky soils, with a light to medium texture, well drained, and 1.5 m depth. As per the occurrence locations relative to elevation (Figure 7), *J. procera* and *O. europaea* subsp. *cuspidata* were found from 1800 to 3200 and 1400 to 3000 m above sea level, respectively. The optimum suitable elevation ranges for *J. procera* and *O. europaea* subsp. *cuspidata*, based on realized niches, were found to be from 2200 to 2600 and 2100 to 2500 m, respectively. These findings are consistent with a study by Bekele‐tesemma (2007), who has reported that *J. procera* performs well in moist and wet mid‐highlands and highland agro‐climatic zones ranging from 1500 to 3300 m, while *O. europaea* does best in moist and wet mid‐highlands and dry highland agro‐climatic zones in all regions, 1400–3100 m above sea level.

### 4.3 Current potential suitability

Our result also emphasized that each of the ecological niche models used in this study gives different results on spatial prediction area. For example, the prediction performance of RF and ANN for *J. procera*, and that of GAM, MAXENT, and RF for *O. europaea* is relatively similar, but there is a difference in their spatial predictions. It can be said that the prediction differences come from the fact that each one of the modeling algorithms starts from different set of assumptions and requirements for data input. Similar to this study, different SDMs may have similar predictive performance (Allouche et al., 2006), yet generate very different predictions of suitable habitat (Beaumont et al., 2016). In several studies, ensemble models have been used as an alternative to reduce variability between the different algorithms, and they have even been proposed as promising techniques for species distribution modeling (Hao et al., 2019; Marmion et al., 2009).

The implications of our findings are crucial for the management and conservation of the target species, leading to benefits for the community. The ensemble suitability maps in this study indicated that the investigated species were significantly restricted in their habitats. The majority of suitable habitat for both *O. europaea* and *J. procera* were concentrated in the highlands of Southern, Eastern, and Western zones of Tigray region. In line to this study, Friis et al. (2010) and Asefa et al. (2020), has confirmed that dry evergreen Afromontane forests, where *J. procera* and *O. europaea* subsp. *cuspidata* are common, covers most of the mountainous topography of the Ethiopian highlands. Ranjitkar et al. (2014), revealed the usefulness of the ensemble forecast technique in describing the climatic comfort zone of an endemic species with restricted distribution. Ensembles are preferred for their superior predictive accuracy, adaptability to diverse ecological factors, and ability to handle data variations. Moreover, ensembles are particularly advantageous in changing environmental conditions and non‐stationarity, enhancing informed decision‐making in conservation, ecology, and resource management contexts. Therefore, this study confirmed the appropriateness of using the ensemble modeling framework, thereby confirming the observations made by Ray and Ramachandra (2017); and Shabani et al. (2016).

## 5 CONCLUSIONS

This study demonstrates that the ensemble model outperformed single models, and it is good enough at predicting the potential suitable habitats of *J. procera* and *O. europaea* tree species with high accuracy. The majority of the current suitable habitats for both species were found to be concentrated in the highlands of the southern, eastern, and western zones of Tigray. Our results also indicated that, about 2721 km^2^ (87%) and 3576 km^2^ (91%) of the total suitable areas for *J. procera* and *O. europaea*, respectively, were found outside the Desa'a and Hugumbirda Grat‐Kahsu state forests, indicating future interventions cannot rely solely on existing protected areas. The high importance of environmental variables in the ensemble output of this study suggests that any future change in the controlling variables would have an impact on the identified potential suitable areas for *J. procera* and *O. europaea*. Therefore, the study recommends that before plantation intervention forestry practitioners should select determinant variables and map out suitable areas using ensemble models (the traditional suitability models require land use requirement data). The target species populations outside the protected areas should be studied, protected and up‐scaled to ensure their conservation as well as to benefit local communities that are rely on them for household income and medicine. As soil information is vital, it is recommended that researchers should focus on mapping soils. We also hypothesized that other locally restricted dry Afromontane tree species may likewise have wider potential suitable habitat, and we suggest applying a similar approach. The impact of future climate change on the studied species through various shared socioeconomic pathways and time periods will be explored in our upcoming paper.

## AUTHOR CONTRIBUTIONS

**Gebreyohannes Zenebe:** Conceptualization (lead); data curation (lead); formal analysis (lead); methodology (lead); validation (equal); writing – original draft (lead). **Amanuel Zenebe:** Conceptualization (equal); methodology (equal); supervision (equal); validation (equal); writing – review and editing (equal). **Emiru Birhane:** Conceptualization (equal); methodology (equal); supervision (equal); validation (equal); writing – review and editing (equal). **Atkilt Girma:** Conceptualization (equal); methodology (equal); supervision (equal); validation (equal); writing – review and editing (equal). **Henok Shiferaw:** Writing – review and editing (equal).

## CONFLICT OF INTEREST STATEMENT

The authors have declared that they have no conflicts of interest.

## Data Availability

The primary data collected during the field work (March to May 2020) will be provided up on request. The other datasets are freely accessed from the following sources: Climatologies at high resolution for the earth's land surface areas (CHELSA) climate from (https://envicloud.wsl.ch/#/?prefix=chelsa%2Fchelsa_V1); ISRIC's SoilGrids 250 m resolution from (https://data.isric.org/); and the SRTM DEM 30 m spatial resolution from (https://earthexplorer.usgs.gov/).

## References

[ece370343-bib-0001] Abebe, D. B. , & Arega, S. (2023). Green Legacy Initiative for sustainable development: POLICY Brief1 . International Conference on Ethiopian Economy. Ethiopian Economics Association (EEA). https://www.researchgate.net/publication/368450412%0AGreen

[ece370343-bib-0002] Abiyu, A. , Teketay, D. , Glatzel, G. , Aerts, R. , & Gratzer, G. (2017). Restoration of degraded ecosystems in the Afromontane highlands of Ethiopia: Comparison of plantations and natural regeneration§. Southern Forests: a Journal of Forest Science, 79(2), 103–108. 10.2989/20702620.2016.1254917

[ece370343-bib-0003] Abrahms, B. , DiPietro, D. , Graffis, A. , & Hollander, A. (2017). Managing biodiversity under climate change: Challenges, frameworks, and tools for adaptation. Biodiversity and Conservation, 26, 2277–2293.

[ece370343-bib-0004] Abrha, H. , Emiru, B. , Haftom, H. , & Ashenafi, M. (2018). Predicting suitable habitats of endangered Juniperus procera tree under climate change in northern Ethiopia. Journal of Sustainable Forestry, 37(8), 842–853. 10.1080/10549811.2018.1494000

[ece370343-bib-0005] Aerts, R. , Van Overtveld, K. , Haile, M. , Hermy, M. , Deckers, J. , & Muys, B. (2006). Species composition and diversity of small Afromontane forest fragments in northern Ethiopia. Plant Ecology, 187, 127–142. 10.1007/s11258-006-9137-0

[ece370343-bib-0006] Allouche, O. , Tsoar, A. , & Kadmon, R. (2006). Assessing the accuracy of species distribution models: Prevalence, kappa and the true skill statistic (TSS). Journal of Applied Ecology, 43(6), 1223–1232. 10.1111/j.1365-2664.2006.01214.x

[ece370343-bib-0007] Asefa, M. , Cao, M. , He, Y. , Mekonnen, E. , Song, X. , & Yang, J. (2020). Ethiopian vegetation types, climate and topography. Plant Diversity, 42, 302–311. 10.1016/j.pld.2020.04.004 33094201 PMC7567763

[ece370343-bib-0008] Aynekulu, E. , Aerts, R. , Moonen, P. , Denich, M. , Gebrehiwot, K. , Vågen, T. G. , Mekuria, W. , & Boehmer, H. J. (2012). Altitudinal variation and conservation priorities of vegetation along the Great Rift Valley escarpment, northern Ethiopia. Biodiversity and Conservation, 21(10), 2691–2707. 10.1007/s10531-012-0328-9

[ece370343-bib-0009] Aynekulu, E. , Denich, M. , & Tsegaye, D. (2009). Regeneration response of *Juniperus procera* and *Olea europaea* subsp *cuspidata* to exclosure in a dry Afromontane Forest in northern Ethiopia. Mountain Research and Development, 29(2), 143–152. 10.1659/mrd.1076

[ece370343-bib-0010] Beaumont, L. J. , Graham, E. , Englert, D. , Wilson, P. D. , Cabrelli, A. , Baumgartner, J. B. , Hallgren, W. , Esperón‐rodríguez, M. , Nipperess, D. A. , Warren, D. L. , Laffan, S. W. , & Vanderwal, J. (2016). Which species distribution models are more (or less) likely to project broad‐scale, climate‐induced shifts in species ranges? Ecological Modelling, 342, 135–146. 10.1016/j.ecolmodel.2016.10.004

[ece370343-bib-0011] Bedia, J. , Busqué, J. , & Gutiérrez, J. M. (2011). Predicting plant species distribution across an alpine rangeland in northern Spain. A comparison of probabilistic methods. Applied Vegetation Science, 14(3), 415–432. 10.1111/j.1654-109X.2011.01128.x

[ece370343-bib-0012] Bekele‐Tesemma, A. (2007). Useful trees and shrubs of Ethiopia: Identification, propagation and management for 17 agroclimatic zones (p. 552) [ B. Tengnäs , E. Kelbesa , S. Demissew , & P. Maundu (Eds.)]. RELMA in ICRAF Project, World Agroforestry Centre—Eastern Africa Region Programme.

[ece370343-bib-0013] Berihu, T. , Girmay, G. , Sebhatleab, M. , & Berhane, E. (2017). Soil carbon and nitrogen losses following deforestation in Ethiopia. Agronomy for Sustainable Development, 37, 1–12. 10.1007/s13593-016-0408-4

[ece370343-bib-0014] Beyene, A. , & Shumetie, A. (2023). Ethiopian Economics Association (EEA). Green Legacy Initiative for sustainable economic development in Ethiopia. Policy Working Paper 10/2023. Augmenting Economic Governance in Ethiopia (AEGE).

[ece370343-bib-0015] Couralet, C. , & Bakamwesiga, H. (2007). Juniperus procera Hochst. Ex Endl. Record from PROTA4U (D. Louppe, A. A. Oteng‐Amoako, & M. Brink, Eds.). PROTA (Plant Resources of Tropical Africa/Ressources végétales de l'Afrique tropicale). http://www.prota4u

[ece370343-bib-0016] Dittrich, A. , Roilo, S. , Sonnenschein, R. , Cerrato, C. , Ewald, M. , Viterbi, R. , & Cord, A. F. (2020). Modelling distributions of rove beetles in mountainous areas using remote sensing data. Remote Sensing, 80(12), 1–24. 10.3390/rs12010080

[ece370343-bib-0017] Engler, R. , Waser, L. T. , Zimmermann, N. , Schaub, M. , Berdos, S. , Ginzler, C. , & Psomas, A. (2013). Combining ensemble modeling and remote sensing for mapping individual tree species at high spatial resolution. Forest Ecology and Management, 310, 64–73.

[ece370343-bib-0018] Fisseha, A. , & Rannestad, M. M. (2022). Challenges and strategy for successful restoration of dry evergreen Afromontane Forests of Ethiopia . https://ssrn.com/abstract=4194276

[ece370343-bib-0019] Friis, I. (1992). Forests and forest trees of northeast tropical Africa: Their natural habitats and distribution patterns in Ethiopia, Djibouti and Somalia. HMSO.

[ece370343-bib-0020] Friis, I. , Demissew, S. , & van Breugel, P. (2010). Atlas of the potential vegetation of Ethiopia (D. K. D. V. Selskab, Ed.). The Royal Danish Academy of Sciences and Letters.

[ece370343-bib-0021] Gastón, A. , García‐Viñas, J. I. , Bravo‐Fernández, A. J. , López‐Leiva, C. , Oliet, J. A. , Roig, S. , & Serrada, R. (2014). Species distribution models applied to plant species selection in forest restoration: Are model predictions comparable to expert opinion? New Forests, 45(5), 641–653. 10.1007/s11056-014-9427-7

[ece370343-bib-0022] Gelashe, B. M. (2017). *Species diversity and distribution patterns of woody plants in Adaba‐Dodola Afromontane Forest, Oromia, Ethiopia* [A dissertation for the degree of doctor of philosophy, Seoul National University].

[ece370343-bib-0023] Giday, K. , Eshete, G. , Barklund, P. , Aertsen, W. , & Muys, B. (2013). Wood biomass functions for *Acacia abyssinica* trees and shrubs and implications for provision of ecosystem services in a community managed exclosure in Tigray, Ethiopia. Journal of Arid Environments, 94, 80–86.

[ece370343-bib-0024] Girma, A. , De Bie, C. A. J. M. , Skidmore, A. K. , Venus, V. , & Bongers, F. (2015). Hyper‐temporal SPOT‐NDVI dataset parameterization captures species distributions. International Journal of Geographical Information Science, 8816, 89–107. 10.1080/13658816.2015.1082565

[ece370343-bib-0025] González‐Ferreras, A. M. , Barquín, J. , & Peñas, F. J. (2016). Integration of habitat models to predict fish distributions in several watersheds of northern Spain. Journal of Applied Ichthyology, 32(1), 204–216. 10.1111/jai.13024

[ece370343-bib-0026] Greenwell, B. , Boehmke, B. , Cunningham, J. , & Developers, G. (2019). gbm: Generalized boosted regression models. R package Version 2.1.5 . https://cran.r‐project.org/package=gbm

[ece370343-bib-0027] Grenouillet, G. , Buisson, L. , Casajus, N. , & Lek, S. (2011). Ensemble modelling of species distribution : The effects of geographical and environmental ranges. Ecography, 34, 9–17. 10.1111/j.1600-0587.2010.06152.x

[ece370343-bib-0028] Gufi, Y. , Manaye, A. , Tesfamariam, B. , Abrha, H. , Gidey, T. , & Gebru, K. M. (2023). Modeling climate change impact on distribution and abundance of Balanites aegyptiaca in drylands of Ethiopia. Modeling Earth Systems and Environment, 9, 3415–3427. 10.1007/s40808-022-01666-2

[ece370343-bib-0029] Hagazi, N. , Gebrekirstos, A. , Birhane, E. , Bongers, F. , Kelly, R. , & Bräuning, A. (2020). Land restoration requires a shift from quantity to quality: Lessons from Tigray, Ethiopia. ETFRN News, 60, 131–138.

[ece370343-bib-0030] Hagos, H. , Abraha, H. , Kelem, G. , & Hadgu, M. (2021). Agroclimatic zonation of Tigray region of Ethiopia based on aridity index and traditional agro‐climatic zones. Journal of Agrometeorology, 21(2), 176–181. 10.54386/jam.v21i2.229

[ece370343-bib-0031] Hao, T. , Elith, J. , Guillera‐Arroita, G. , & Lahoz‐Monfort, J. J. (2019). A review of evidence about use and performance of species distribution modelling ensembles like BIOMOD. Diversity and Distributions, 25(5), 839–852. 10.1111/ddi.12892

[ece370343-bib-0032] Hastie, T. (2019). *gam: Generalized additive models*. R package Version 1.16.1. https://cran.r‐project.org/package=gam

[ece370343-bib-0033] Hengl, T. , De Jesus, J. M. , Heuvelink, G. B. M. , Gonzalez, M. R. , Kilibarda, M. , Blagotić, A. , Shangguan, W. , Wright, M. N. , Geng, X. , Bauer‐Marschallinger, B. , Guevara, M. A. , Vargas, R. , MacMillan, R. A. , Batjes, N. H. , Leenaars, J. G. B. , Ribeiro, E. , Wheeler, I. , Mantel, S. , & Kempen, B. (2017). SoilGrids250m: Global gridded soil information based on machine learning. PLoS One, 12(2), 1–40. 10.1371/journal.pone.0169748 PMC5313206 28207752

[ece370343-bib-0034] Hijmans, R. J. , Phillips, S. , Leathwick, J. , & Elith, J. (2017). *dismo: Species distribution modeling*. R package Version 1.1‐4. https://cran.r‐project.org/package=dismo

[ece370343-bib-0035] Hishe, H. , Giday, K. , & Haile, M. (2015). The influence of physical factors on deforestation of key species and their implication for forest management in the dry Afromontane Forest of Desa'a, northern Ethiopia. International Journal of Science and Research, 4(3), 2400–2407.

[ece370343-bib-0036] Hishe, H. , Oosterlynck, L. , Giday, K. , De Keersmaecker, W. , Somers, B. , & Muys, B. (2021). A combination of climate, tree diversity and local human disturbance determine the stability of dry Afromontane forests. Forest Ecosystems, 8, 16. 10.1186/s40663-021-00288-x

[ece370343-bib-0037] James, G. , Witten, D. , Hastie, T. , & Tibshirani, R. (2013). An introduction to statistical learning—With applications in R, springer texts in statistics (G. Casella, S. Fienberg, & I. Olkin, Eds.). Springer Science+Business Media. 10.1007/978-1-4614-7138-7

[ece370343-bib-0038] Jones, S. (2015). African Mountain Forests – Current satatus, critical issues, and ways forward for conservation. In S. Jones (Ed.), Drylands Coordination Group Proceedings No. 26 (pp. 1–38). Drylands Coordination Group Proceedings.

[ece370343-bib-0039] Karger, D. N. , Conrad, O. , Böhner, J. , Kawohl, T. , Kreft, H. , Soria‐Auza, R. W. , Zimmermann, N. E. , Linder, H. P. , & Kessler, M. (2017). Climatologies at high resolution for the earth's land surface areas. Scientific Data, 4(September), 1–20. 10.1038/sdata.2017.122 PMC5584396 28872642

[ece370343-bib-0040] Khan, S. , & Verma, S. (2022). Ensemble modeling to predict the impact of future climate change on the global distribution of *Olea europaea* subsp. *cuspidata* . Frontiers in Forests and Global Change, 5, 977691. 10.3389/ffgc.2022.977691

[ece370343-bib-0041] Kindt, R. (2018). Ensemble species distribution modelling with transformed suitability values. Environmental Modelling and Software, 100, 136–145. 10.1016/j.envsoft.2017.11.009

[ece370343-bib-0042] Kindt, R. , & Coe, R. (2005). Tree diversity analysis. A manual and software for common statistical methods for ecological and biodiversity studies. World Agroforestry Centre (ICRAF). http://www.worldagroforestry.org/output/tree‐diversity‐analysis

[ece370343-bib-0043] Liaw, A. , & Wiener, M. (2002). Classification and regression by randomForest. R News, 2(3), 18–22. https://cran.r‐project.org/doc/Rnews/

[ece370343-bib-0044] Marmion, M. , Parviainen, M. , Luoto, M. , & Heikkinen, R. K. (2009). Evaluation of consensus methods in predictive species distribution modelling. Diversity and Distributions, 15, 59–69. 10.1111/j.1472-4642.2008.00491.x

[ece370343-bib-0045] Mcintyre, S. , Rangel, E. F. , Ready, P. D. , & Carvalho, B. M. (2017). Species‐specific ecological niche modelling predicts different range contractions for *Lutzomyia intermedia* and a related vector of *Leishmania braziliensis* following climate change in South America. Parasites & Vectors, 10(157), 1–15. 10.1186/s13071-017-2093-9 28340594 PMC5366140

[ece370343-bib-0046] MEFCC . (2017). National Forest Sector Development Program, Ethiopia (Vol. III). Synthesis report . https://www.et.undp.org/content/dam/ethiopia/docs/2018/National

[ece370343-bib-0047] Montoya‐Jiménez, J. C. , Valdez‐Lazalde, J. R. , Ángeles‐Perez, G. , de los SantosPosadas, H. M. , & Cruz‐Cárdenas, G. (2022). Predictive capacity of nine algorithms and an ensemble model to determine the geographic distribution of tree species. iForest, 15, 363–371. 10.3832/ifor4084-015

[ece370343-bib-0048] Naimi, B. , Hamm, N. A. , Groen, T. A. , Skidmore, A. K. , & Toxopeus, A. G. (2014). Where is positional uncertainty a problem for species distribution modelling. Ecography, 37, 191–203. 10.1111/j.1600-0587.2013.00205.x

[ece370343-bib-0049] Negash, E. , Birhane, E. , Gebrekirstos, A. , Gebremedhin, M. A. , Annys, S. , Rannestad, M. M. , Berhe, D. H. , Sisay, A. , Alemayehu, T. , Berhane, T. , Gebru, B. M. , Solomon, N. , & Nyssen, J. (2023). Remote sensing reveals how armed conflict regressed woody vegetation cover and ecosystem restoration efforts in Tigray (Ethiopia). Science of Remote Sensing, 8(August), 100108. 10.1016/j.srs.2023.100108

[ece370343-bib-0050] Nzuva, S. M. , & Nderu, L. (2019). The superiority of the ensemble classification methods: A comprehensive review. Journal of Information Engineering and Applications, 19(5), 43–53. 10.7176/jiea/9-5-05

[ece370343-bib-0051] O'Donnell, M. S. , & Ignizio, D. A. (2012). Bioclimatic predictors for supporting ecological applications in the conterminous United States. U.S Geological Survey Data Series, 691, 10.

[ece370343-bib-0052] Ourge, M. , Hofstad, O. , Klanderud, K. , Eldegard, K. , & Tewolde‐Berhan, S. (2018). Illegal harvesting of locally endangered *Olea europaea* Subsp. *cuspidata* (Wall. ex G. Don) Cif. and its causes in Hugumburda Forest, Northern Ethiopia. Forests, 9(498), 1–13. 10.3390/f9080498

[ece370343-bib-0053] Porfirio, L. L. , Harris, R. M. B. , Lefroy, E. C. , Hugh, S. , Gould, S. F. , Lee, G. , Bindoff, N. L. , & Mackey, B. (2014). Improving the use of species distribution models in conservation planning and management under climate change. PLoS One, 9(11), 1–21. 10.1371/journal.pone.0113749 PMC4242662 25420020

[ece370343-bib-0054] R Core Team . (2019). R: A language and environment for statistical computing. R Foundation for Statistical Computing. https://www.r‐project.org/

[ece370343-bib-0055] Ranjitkar, S. , Xu, J. , & Shrestha, K. K. (2014). Ensemble forecast of climate suitability for the trans‐Himalayan Nyctaginaceae species. Ecological Modelling, 282, 18–24. 10.1016/j.ecolmodel.2014.03.003

[ece370343-bib-0056] Ray, R. , & Ramachandra, T. (2017). Optimization of ensemble modeling approach for studying climatic Nich and conservation status assessment for endemic taxa. International Journal of Ecology & Development, 32(1), 18–32.

[ece370343-bib-0057] Reubens, B. , Moeremans, C. , Poesen, J. , Nyssen, J. , Tewoldeberhan, S. , Franzel, S. , Deckers, J. , Orwa, C. , & Muys, B. (2011). Tree species selection for land rehabilitation in Ethiopia: From fragmented knowledge to an integrated multi‐criteria decision approach. Agroforestry Systems, 82(3), 303–330. 10.1007/s10457-011-9381-8

[ece370343-bib-0058] Shabani, F. , Kumar, L. , & Ahmadi, M. (2016). A comparison of absolute performance of different correlative and mechanistic species distribution models in an independent area. Ecology and Evolution, 6(16), 5973–5986. 10.1002/ece3.2332 27547370 PMC4983607

[ece370343-bib-0059] Shrestha, U. B. , & Bawa, K. S. (2014). Impact of climate change on potential distribution of Chinese caterpillar fungus (*Ophiocordyceps sinensis*) in Nepal Himalaya. PLoS One, 9(9), 1–11. 10.1371/journal.pone.0106405 PMC4152242 25180515

[ece370343-bib-0060] Siyum, Z. G. , Onilude, J. O. A. M. A. , & Tolera, M. (2019). Growth trajectories and ages of main tree species in dry Afromontane forest fragments of northern Ethiopia. SN Applied Sciences, 1(7), 1–17. 10.1007/s42452-019-0803-y

[ece370343-bib-0061] Solomon, N. , Pabi, O. , Annang, T. , Asante, I. K. , & Birhane, E. (2018). The effects of land cover change on carbon stock dynamics in a dry Afromontane forest in northern Ethiopia. Carbon Balance and Management, 13, 14. 10.1186/s13021-018-0103-7 30191432 PMC6127076

[ece370343-bib-0062] Takele, A. , Lakew, H. B. , & Kabite, G. (2022). Does the recent afforestation program in Ethiopia influenced vegetation cover and hydrology? A case study in the upper awash basin, Ethiopia. Heliyon, 8(6), e09589. 10.1016/j.heliyon.2022.e09589 35669547 PMC9163509

[ece370343-bib-0063] Thuiller, W. , Albert, C. , Araújo, M. B. , Berry, P. M. , Cabeza, M. , Guisan, A. , Hickler, T. , Midgley, G. F. , Paterson, J. , Schurr, F. M. , Sykes, M. T. , & Zimmermann, N. E. (2008). Predicting global change impacts on plant species' distributions: Future challenges. Perspectives in Plant Ecology, Evolution and Systematics, 9(3–4), 137–152. 10.1016/j.ppees.2007.09.004

[ece370343-bib-0064] Tigabu, M. , Fjellström, J. , Odén, P. C. , & Teketay, D. (2007). Germination of Juniperus procera seeds in response to stratification and smoke treatments, and detection of insect‐damaged seeds with VIS+ NIR spectroscopy. New Forests, 33, 155–169. 10.1007/s11056-006-9020-9

[ece370343-bib-0065] Tura, T. , Soromessa, T. , Leta, S. , & Argaw, M. (2017). Plant community composition and structure of asabot dry afromontane. Journal of Biodiversity & Endangered Species, 5(4), 1–12. 10.4172/2332-2543.1000202

[ece370343-bib-0066] van der Vossen, H. A. , Mashungwa, G. , & Mmolotsi, R. (2007). Olea europaea L. Record from PROTA4U (H. A. M. van der Vossen, & G.S. Mkamilo, Eds.). PROTA (Plant Resources of Tropical Africa/Ressources végétales de l'Afrique tropicale). http://www.prota4u.org/search.asp

[ece370343-bib-0067] Venables, W. N. , & Ripley, B. D. (2002). Modern applied statistics with S (4th ed.). Springer. http://www.stats.ox.ac.uk/pub/MASS4

[ece370343-bib-0068] Walthert, L. , & Meier, E. S. (2017). Tree species distribution in temperate forests is more influenced by soil than by climate. Ecology and Evolution, 7, 9473–9484. 10.1002/ece3.3436 29187983 PMC5696420

[ece370343-bib-0069] Wang, C. , Wan, J. , Mu, X. , & Zhang, Z. (2015). Management planning for endangered plant species in priority protected areas. Biodiversity and Conservation, 24(10), 2383–2397. 10.1007/s10531-015-0928-2

[ece370343-bib-0070] Wang, T. , Dan, Y. , Jiangfeng, L. , & Keping, M. (2003). Advances in research on the relationship between climatic change and tree‐ring width. Acta Phytoecological Sinica, 27(1), 23–33.

[ece370343-bib-0071] Wani, I. A. , Khan, S. , Verma, S. , Al‐Misned, F. A. , Shafik, H. M. , & El‐Serehy, H. A. (2022). Predicting habitat suitability and niche dynamics of *Dactylorhiza hatagirea* and *Rheum webbianum* in the Himalaya under projected climate change. Scientific Reports, 12(1), 13205. 10.1038/s41598-022-16837-5 35915126 PMC9343649

[ece370343-bib-0072] Wegasie, M. O. , Klanderud, K. , Totland, Ø. , & Eldegard, K. (2021). Ontogenetic niche shifts in a locally endangered tree species (*Olea europaea* subsp. *cuspidata*) in a disturbed forest in northern Ethiopia: Implications for conservation. PLoS One, 16(9), e0256843. 10.1371/journal.pone.0256843 34591856 PMC8483397

[ece370343-bib-0073] Woldemichael, L. , Bekele, T. , & Nemomissa, S. (2010). Vegetation composition in Hugumbirda‐Gratkhassu National Forest Priority Area, South Tigray. Momona Ethiopian Journal of Science, 2(2), 2–48. 10.4314/mejs.v2i2.57673

[ece370343-bib-0074] Worku, E. , & Soromessa, T. (2015). Allometric equation for biomass determination in *Juniperus procera* Endl. and *Podocarpus falcatus* Mirb of Wof‐Washa Forest: Implication for climate change mitigation. American Journal of Life Sciences, 3(3), 190–202. 10.11648/j.ajls.20150303.20

[ece370343-bib-0075] Worthington, T. A. , Zhang, T. , Logue, D. R. , Mittelstet, A. R. , & Brewer, S. K. (2016). Landscape and flow metrics affecting the distribution of a federally‐threatened fish: Improving management, model fit, and model transferability. Ecological Modelling, 342, 1–18. 10.1016/j.ecolmodel.2016.09.016

[ece370343-bib-0076] Young, N. E. , Romme, W. H. , Evangelista, P. H. , Mengistu, T. , & Worede, A. (2017). Variation in population structure and dynamics of montane forest tree species in Ethiopia guide priorities for conservation and research. Biotropica, 49, 309–317. 10.1111/btp.12420

